# The Roles of Pre–P-wave Versus Peri–P-wave Fractionated Electrograms for Atrial Substrate Beyond Entrainment Response

**DOI:** 10.19102/icrm.2023.14065

**Published:** 2023-06-15

**Authors:** Tolga Han Efe, Idriz Merovci, Fatih Oksuz, Erdeniz Eris, Meryem Kara, Duygu Kocyigit Burunkaya, Elif Hande Ozcan Cetin, Ahmet Korkmaz, Ozcan Ozeke, Serkan Cay, Firat Ozcan, Dursun Aras, Serkan Topaloglu

**Affiliations:** ^1^Department of Cardiology, University of Health Sciences, Dıs¸kapı Yıldırım Beyazıt Training and Research Hospital, Ankara, Turkey; ^2^Department of Cardiology, University Clinical Center of Kosovo, Prishtina, Kosovo; ^3^Department of Cardiology, University of Health Sciences, Ankara Hospital, Ankara, Turkey; ^4^Department of Cardiology, University of Health Sciences, Ankara City Hospital, Ankara, Turkey; ^5^Department of Cardiology, I˙stanbul Medipol University, Istanbul, Turkey

**Keywords:** 2:1 intra-atrial block, atrial tachycardia, double tachycardia, dual loop, fragmentation, multiple loops

## Abstract

Atrial tachycardia (AT) with alternating cycle lengths is sparsely reported, and, hence, the ideal mapping strategy has not been firmly established. Beyond the entrainment during tachycardia, some fragmentation characteristics might also give important clues for its possible participation in the macro–re-entrant circuit. We discuss a patient with prior atrial septal defect surgical closures who presented with dual macro–re-entrant ATs related to a fragmented area on the right atrial free wall (240 ms) and the cavotricuspid isthmus (260 ms), respectively. After ablation of the fastest AT on the lateral right atrial free wall, the cycle of the first AT changed to the second AT that was interrupted on cavotricuspid isthmus, proving the dual tachycardia mechanism. This case report addresses the utilization of electroanatomic mapping information as well as fractionated electrogram timing with respect to the surface P-wave as guides for ablation location.

## Case presentation

**Figure 1:**
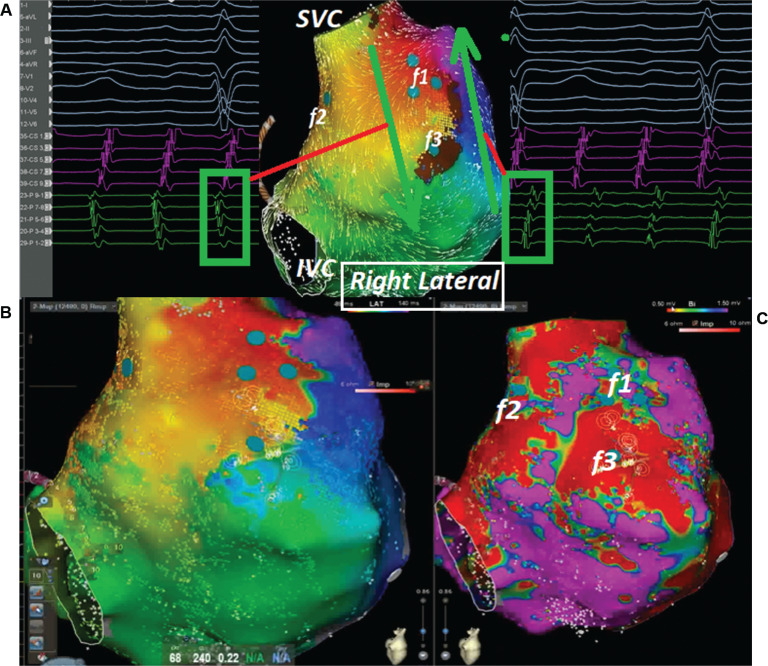
The opposite side activation sequence seen in the anterior and posterior sides of the right atrial free wall **(A)** in activation **(B)** and voltage maps **(C)** with fragmented signal regions (f1, f2, and f3). *Abbreviations:* IVC, inferior vena cava; SVC, superior vena cava.

**Figure 2:**
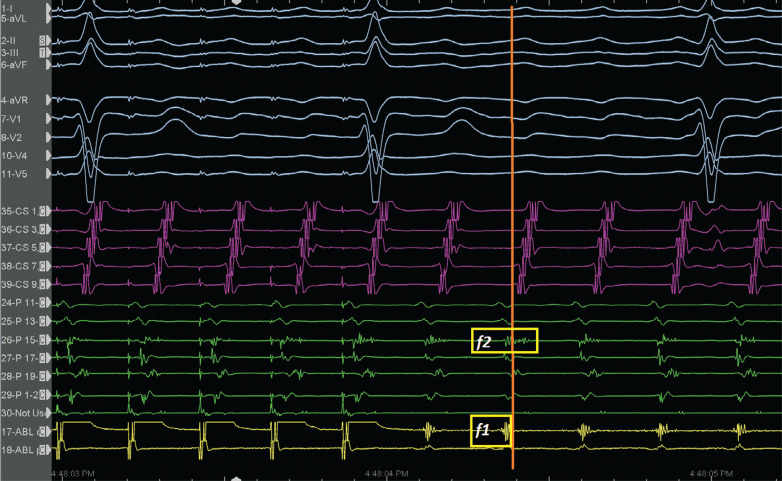
The relationship of f1 and f2 fragmented signals with P-wave onset (orange line). While f1 is seen as pre-P, f2 signals are seen as peri-P. The entrainment response from the f1 region is also seen. The orange line corresponds to the beginning of the P-waves.

**Figure 3:**
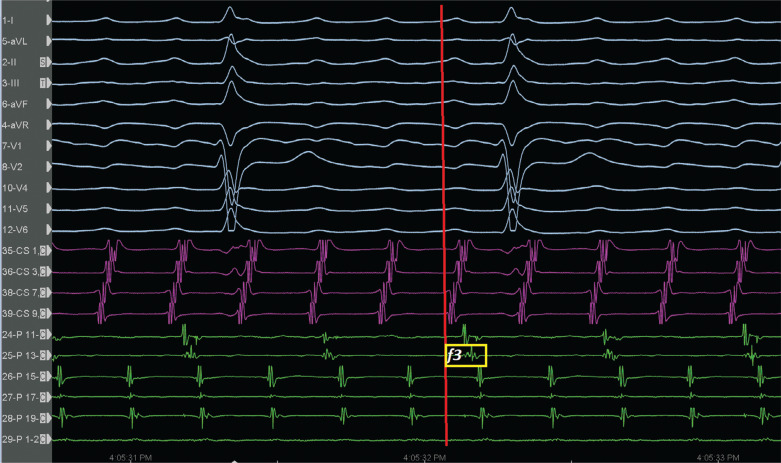
The relationship of f3 fragmented signals with P-wave onset (red line). Note also 2:1 intra-atrial block at the f3 region. The red line corresponds to the beginning of the P-waves.

**Figure 4:**
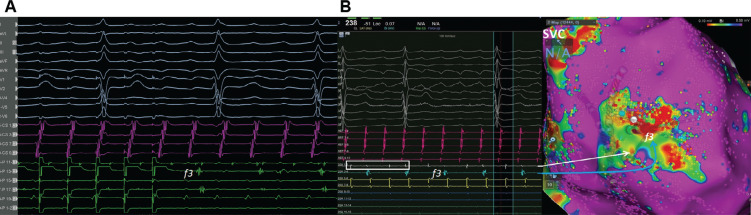
Note 2:1 intra-atrial block at the f3 region **(A)** and irregularly regular atrial alternans (white rectangle in **B**) with alternating double potentials.

**Figure 5:**
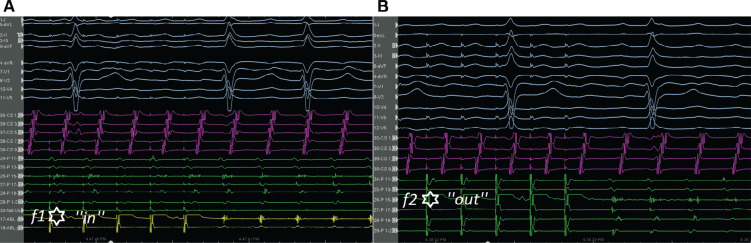
Entrainment responses show that f1 was an in-the-circuit pacing site **(A)**, whereas f2 was an out-of-the-circuit pacing site **(B)**.

**Figure 6:**
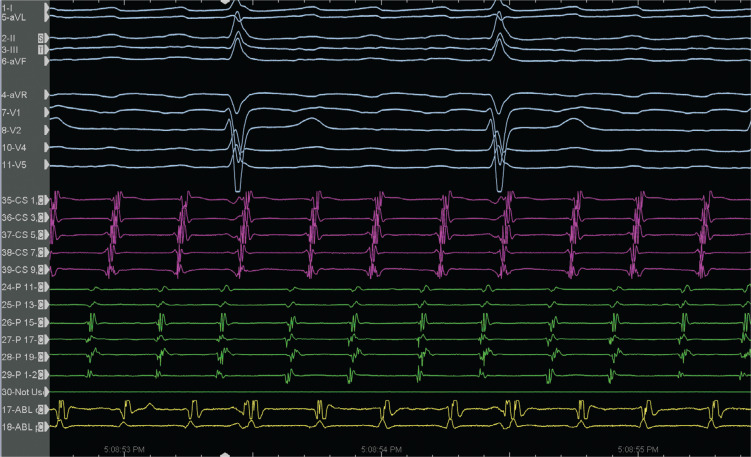
Atrial tachycardia with alternating cycle lengths is seen (alternating 240-ms and 260-ms cycle lengths).

A 65-year-old male patient with a history of previous surgical atrial septal defect closure presented with incessant atrial tachycardia (AT) and was referred for an electrophysiological study. An electrocardiogram showed positive flutter waves in the inferior leads but was negative in lead V1 with a tachycardia cycle length (CL) of 240 ms (CL-1). The considerably long post-pacing interval (PPI) minus the tachycardia CL in combination with the proximal-to-distal activation pattern in the coronary sinus supported the right atrial (RA) origin of the AT. As a long PPI was also detected from the cavotricuspid isthmus (CTI) region and the opposite-side activation sequence in the anterior and posterior sides of the RA free wall (RAFW; **Figure 1A**), 3-dimensional electroanatomic mapping (EAM) (CARTO III Software Version 7, CARTO Prime; Biosense Webster, Diamond Bar, CA, USA) was performed in the right atrium. In the acquired map, we noticed 3 different fragmented signals during tachycardia in different regions (f1, f2, and f3 in **Figure 1**). While the first fragmented signal (f1) was located at the upper side of the RAFW **(Figures 1 and 2 and Video 1)**, the second (f2) was detected in the posterior side of the superior vena cava **(Figures 1 and 2 and Video 2)**, and the last one (f3) was at the middle of the RAFW **(Figures 1, 3, and 4 and Video 3)**. The tachycardia continued but without activation at the site of the recording electrodes (as seen in **Figures 3 and 4**), suggesting that the f3 site was not critical for the tachycardia circuit. Furthermore, the PPI values showed that f1 was an in-the-circuit pacing site, whereas f2 was an out-of-the-circuit pacing site **(Figure 5)**. Therefore, we considered that the tachycardia circle was a classical incisional intra-atrial re-entrant tachycardia originating in the RAFW **(Video 4)**. Interestingly, an attempt was made to entrain atrial flutter by pacing from the RAFW, converting it to an AT with alternating CLs of 240 ms (CL-1) and 260 ms (CL-2) **(Figure 6)**. As the coherent **(Video 5)** and propagation **(Video 6)** maps showed figure-of-8 activation patterns, no additional entrainment attempt was performed. Ablation from the f1 site gradually converted CL-1 (240 ms) to CL-2 (260 ms) before termination of the tachycardia by CTI ablation. We questioned where any clue about fragmentation characteristics exists beyond the PPI evaluation.

## Discussion

Stable beat-to-beat CL variability (CL alternans) in AT has been sparsely reported,^1–5^ especially in complicated tachycardia.^1,2,4,6–8^ Sometimes a tachycardia may initiate another or occur simultaneously. When the faster index AT is running, the index AT repetitively resets the second longer circuit in each beat.^9^ Takigawa et al. found that 16.7% of cases had CL variability in their multiloop AT group, and this CL variability was more frequently observed in macro–re-entrant ATs.^10^ Small re-entrant circuits or localized re-entry is typified by very slow conduction, resulting in long-duration fragmented electrograms (EGMs) at the critical site. Frontera et al. argued that re-entrant ATs have multiple sequential isthmuses of slow conduction, serving as sequential isthmuses rather than a single isthmus.^8^ One AT focus is allowed to depolarize when the other AT conduction occurs over the slower isthmus conduction, and, if these tachycardias have similar intra-atrial activation sequences, the situation can be very challenging. However, the present case highlights several important points.

First, our case emphasizes that it is important to be alert to the possibility of double tachycardia during the ablation procedure as a switch from a certain tachycardia to another creates confusion **(Figure 6)**.^11^ The EAM system has an opportunity for simultaneous activation mappings under 2 different CLs using a parallel mapping system; additionally, the threshold for stable CL criteria or parallel mapping during mapping can be adjusted to focus on the target CL, resulting in separated and accurate maps for long and short CLs, respectively.^12–14^

Second, the fragmentation characteristics might give us important clues about its participation in tachycardia beyond the entrainment attempts. These fractionated signals represent underlying slow conduction caused by impaired intercellular coupling, which results in the potential for micro– or macro–re-entry. Discerning macro–re-entrant from centrifugal arrhythmias is more than an academic exercise.^15^ Postsurgical atrial isthmuses are complex structures, with multiple entrances, exits, and dead ends of activation; therefore, the presence of the multiple fractionations might lead to multiple “wannabe re-entries” and incorrect ablation as well.^16^ However, the EGM fractionation covering a great proportion of the CL of AT within a small area guides ablation in localized micro–re-entry.^17^ On the other hand, for macro–re-entrant circuits lacking an isoelectric interval, delineation of the complete circuit path is necessary to choose the target isthmus. The high-resolution mapping may provide a tailored ablation to the most practical and vulnerable isthmus.^18^ Dynamic voltage attenuation studies suggest that a single binary threshold may not exist, which can accurately characterize atrial fibrosis.^19^ Therefore, dynamically adjusting voltage thresholds can identify heterogeneous atrial scarring more accurately (compare **Figure 1C** and **Figure 4B** voltage mappings). Of course, the distinct recording of alternate fractionated potentials by the EAM with high-density catheters^5,12^ is important to evaluate the complex atrial substrate. Without careful recognition of these fragmentation characteristics, operators may be misled to deliver ablation at these pseudo–re-entrant sites irrelevant to the clinical tachycardia. As the wavefront leaves the isthmus at the exit from which the more normal atrial myocardium is rapidly depolarized, which results in the formation of the P-waves, it is expected to see these fragmented signals as “pre–P-waves” for the critical isthmus as the activation of the exit site corresponds to the onset of the P-wave on the surface electrocardiogram.^15,20^ Therefore, if the EGM at the “early” site is not presystolic, it is unlikely that this site is responsible for the AT^15^ **(Figure 2)**. In the current case, both the f2 and f3 signals were likely bystander fractionations, as they extended beyond the onset of the P-wave^16^ **(Figures 2 and 3)**. It should be noted that these mechanistic clues can only be helpful if the P-wave can be characterized without superimposition of the T-wave or the QRS.^15^ Given the risk of changing or terminating the tachycardia that might be difficult to induce, these fragmentation characteristics also prevent unnecessary entrainment attempts during mapping.^21^

**Figure 7:**
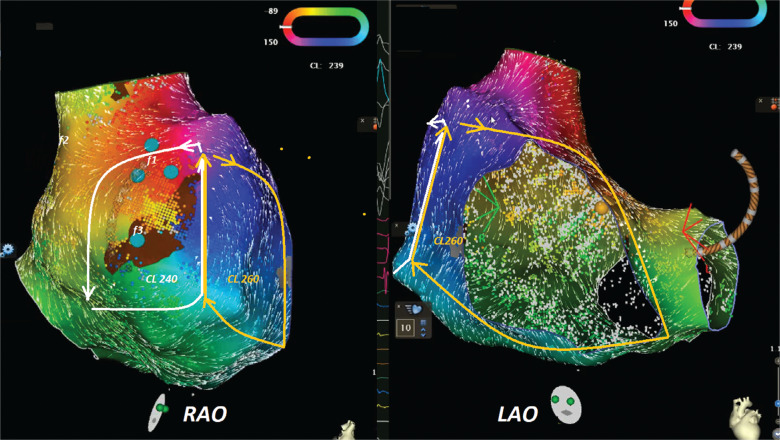
A figure-of-8 activation pattern is seen. *Abbreviations:* LAO, left anterior oblique; RAO, right anterior oblique.

Third, the complex ablation lesions in patients with prior valve surgery, prior ablation, or atrial myopathy may result in unintended localized conduction block or compartmentalized areas in the right atrium,^22^ as seen in the f3 site with 2:1 intra-atrial block **(Figures 3 and 4 and Video 3)**.^23^ Avoiding complete interval lines in conjunction with CTI and septal ablation seems prudent, especially if there is a pre-existing scar in this region.^22^ This is important because even limited ablation has the potential to interrupt conduction to the atrioventricular node in patients with extensive fibrosis of the right atrium.^22–26^ Therefore, despite the detection of the common isthmus in the current case **(Figure 7)**, we preferred to ablate the f1 area and CTI line separately.

## Figures and Tables

**Video 1:** Fragmented signals and entrainment response are seen in the f1 region.

**Video 2:** Fragmented signals and entrainment response are seen in the f2 region.

**Video 3:** A 2:1 intra-atrial block and irregularly regular atrial alternans are seen in the f3 region.

**Video 4:** Coherent mapping shows the figure-of-8 re-entry.

**Video 5:** Propagation mapping shows the figure-of-8 re-entry.

**Video 6:** A gradual cycle length prolongation (from 240 to 260 ms) by ablation from the f1 site.

## References

[1] O’Neill MD, Hocini M, Matsuo S, Haissaguerre M (2007). Twin perimitral atrial flutters with alternating cycle lengths. J Cardiovasc Electrophysiol.

[2] Zhou D, Hu W, Yang G (2020). A postsurgery atrial tachycardia with alternating cycle length: the possible circuits revealed by high-resolution mapping. Heart Rhythm Case Rep.

[3] Ito T, Kaneko Y, Taniguchi Y (2000). Long RP tachycardia with RR alternation. Pacing Clin Electrophysiol.

[4] Nakashima D, Kojima S, Nakaboh A (2008). A atrial flutter consisted of two different conduction pathways. Int J Cardiol.

[5] Su C, Liu M, Ma Y, Wang L (2019). Tachycardia cycle length alternans caused by alternate conduction velocity on the slow conduction zone in a postoperative scar-related atrial tachycardia. J Arrhythm.

[6] Lim PB, Wright IJ, Salukhe TV, Lefroy DC (2011). Narrow complex tachycardia with alternating cycle length: what is the mechanism?. J Cardiovasc Electrophysiol.

[7] John WS, Win-Kuang S, Komandoor SS (2015). Atrial tachycardia with fluctuating cycle length post multiple atrial fibrillation ablations.

[8] Frontera A, Mahajan R, Dallet C (2019). Characterizing localized reentry with high-resolution mapping: evidence for multiple slow conducting isthmuses within the circuit. Heart Rhythm.

[9] Takigawa M, Derval N, Martin CA (2019). A simple mechanism underlying the behavior of reentrant atrial tachycardia during ablation. Heart Rhythm.

[10] Takigawa M, Derval N, Maury P (2018). Comprehensive multicenter study of the common isthmus in post-atrial fibrillation ablation multiple-loop atrial tachycardia. Circ Arrhythm Electrophysiol.

[11] Soejima K (2001). Double trouble in the electrophysiology laboratory. J Cardiovasc Electrophysiol.

[12] Zhang J, Zheng L, Zhou D (2019). Insight into the mechanism of macroreentrant atrial tachycardia with cycle length alternans using ultrahigh density mapping system. Circ Arrhythm Electrophysiol.

[13] Nies M, Schleberger R, Dinshaw L, Rillig A, Metzner A, Meyer C (2021). Parallel mapping and catheter ablation of polymorphic premature ventricular contractions: a new feature for activation mapping. Case Rep Cardiol.

[14] Massoullie G, Schmitt B, Andronache M (2020). Biatrial mapping of two simultaneous tachycardia using parallel mapping module. Europace.

[15] Chugh A (2021). Mapping and ablation of post-AF atrial tachycardias. J Cardiovasc Electrophysiol.

[16] Luther V, Sikkel M, Bennett N (2017). Visualizing localized reentry with ultra-high density mapping in iatrogenic atrial tachycardia: beware pseudo-reentry. Circ Arrhythm Electrophysiol.

[17] Jais P, Matsuo S, Knecht S (2009). A deductive mapping strategy for atrial tachycardia following atrial fibrillation ablation: importance of localized reentry. J Cardiovasc Electrophysiol.

[18] Takigawa M, Derval N, Frontera A (2018). Revisiting anatomic macroreentrant tachycardia after atrial fibrillation ablation using ultrahigh-resolution mapping: implications for ablation. Heart Rhythm.

[19] Methachittiphan N, Akoum N, Gopinathannair R, Boyle PM, Sridhar AR (2020). Dynamic voltage threshold adjusted substrate modification technique for complex atypical atrial flutters with varying circuits. Pacing Clin Electrophysiol.

[20] Rav-Acha M, Ng CY, Heist EK (2016). A novel annotation technique during mapping to facilitate the termination of atrial tachycardia following ablation for atrial fibrillation. J Cardiovasc Electrophysiol.

[21] Ma W, Qiu J, Lu F (2021). Catheter ablation for atrial tachycardias: how to interpret the unclear activation map?. Pacing Clin Electrophysiol.

[22] Markowitz SM, Choi DY, Daian F (2019). Regional isolation in the right atrium with disruption of intra-atrial conduction after catheter ablation of atrial tachycardia. J Cardiovasc Electrophysiol.

[23] Kofune M, Watanabe I, Okumura Y (2015). Right atrial tachycardia with 2:1 intra-atrial conduction. J Arrhythm.

[24] Ali H, Sorgente A, Epicoco G, Cappato R (2015). An uncommon block of a common circuit. J Cardiovasc Electrophysiol.

[25] Chugh A, Yokokawa M, Baman T, Bogun F, Wu A (2012). Intra-atrial conduction block mimicking atrioventricular nodal block after multiple catheter ablation procedures for atrial tachycardia in a patient with cardiomyopathy. J Cardiovasc Electrophysiol.

[26] Nakatani Y, Krisai P, Nakashima T (2021). Atrioventricular block with coronary sinus potential dissociation after lateral mitral isthmus block: what is the mechanism?. J Cardiovasc Electrophysiol.

